# P04.24. Use of relaxation techniques and complementary and alternative medicine by adults with insomnia symptoms: results from a national survey

**DOI:** 10.1186/1472-6882-12-S1-P294

**Published:** 2012-06-12

**Authors:** S Bertisch, R Erwin Wells, M Smith, E McCarthy

**Affiliations:** 1 Beth Israel Deaconess Medical Center, Harvard Medical School, Boston, USA; 2 Brigham and Women's Hospital, Boston, USA; 3 Johns Hopkins Bayview Medical Center, Baltimore, USA

## Purpose

An estimated 1.6 million U.S. adults use complementary and alternative medicine (CAM) for insomnia. The American Academy of Sleep Medicine has graded relaxation training as a “Standard” treatment for insomnia. However, national patterns of CAM and relaxation techniques by American adults with insomnia are not well-established. In this context, we sought to identify overall rates of CAM and relaxation technique use, determine correlates of relaxation technique use, and to quantify reasons for use and disclosure of CAM and relaxation techniques to conventional medical professionals.

## Methods

We analyzed data from the 2007 National Health Interview Survey (n=23,358). We estimated prevalence of CAM and relaxation techniques use among adults by self-reported insomnia symptom status. Among respondents with insomnia symptoms (n=4,415), we examined reasons for use and disclosure to medical professionals. We employed multivariable logistic regression to determine the association between relaxation technique use and insomnia symptoms, adjusting for potential confounders.

## Results

Nearly 50% of adults with insomnia symptoms use CAM annually. Twenty-six percent of adults with insomnia symptoms, an estimated 10.8 million, use relaxation techniques annually, and have higher likelihood of use compared with adults without insomnia (aOR 1.48, 95% CI 1.32,1.66). Deep breathing exercises are the most common relaxation technique used by adults with insomnia symptoms. Despite high rates of use of CAM and relaxation techniques, use specifically for treatment of insomnia was uncommon. Only 25% of adults with insomnia symptoms disclosed their relaxation techniques use to medical professionals. Age ≥ 70 years, being male, lower educational attainment, low physical activity, and living in the South were associated with lower relaxation technique use among adults with insomnia.

## Conclusion

Adults with insomnia symptoms commonly use relaxation techniques and CAM, yet use specifically for treatment of insomnia is low. Increased provider-patient communication regarding the benefits of relaxation techniques for insomnia may facilitate more targeted use.

